# Glomerular Capillary C4d Staining in Native Kidney Biopsies: Associations with Proteinuric Phenotype and Reduced Likelihood of Early Proteinuria Remission

**DOI:** 10.3390/diagnostics16172717

**Published:** 2026-08-26

**Authors:** Mehmet Sezen, Melike Nalbant, Suat Akgür, Ayşe Şeker, Nermin Ünal Odabaş, Sibel Bayer, Türker Emre, Mehmet Usta, Yavuz Ayar

**Affiliations:** 1 Nephrology Clinic, Bursa City Hospital, Bursa 16110, Türkiye; doctrayse@gmail.com (A.Ş.); aturkeremre@yahoo.com.tr (T.E.); dr.mehmet.usta@hotmail.com (M.U.); yavuzayar@hotmail.com (Y.A.); 2 Department of Pathology, Bursa City Hospital, Bursa 16110, Türkiye; drmelike@gmail.com (M.N.); drnerminunal@gmail.com (N.Ü.O.); sibelbayer@gmail.com (S.B.); 3 Nephrology Clinic, Bursa Çekirge State Hospital, Bursa 16090, Türkiye; suatakgur@yahoo.com

**Keywords:** glomerular diseases, native kidney biopsy, C4d, prognosis

## Abstract

**Background/Objectives**: This study investigated the prevalence and clinical significance of glomerular capillary C4d staining in native kidney biopsies and its association with renal outcomes in patients with biopsy-proven glomerular diseases. **Methods**: This single-center, retrospective study reviewed adult native kidney biopsy cases performed between November 2022 and November 2024 and included 173 patients with biopsy-proven glomerular disease who underwent immunohistochemical C4d staining. **Results**: C4d positivity was detected in 38.2% of biopsies, most frequently in membranous nephropathy (74.2%), followed by AA amyloidosis (63.6%) and lupus nephritis (63.6%). General characteristics were similar between C4d-negative and C4d-positive patients; however, the C4d-positive group had higher baseline LDL and proteinuria levels and lower albumin levels. At 6 months, the proportion of patients who achieved proteinuria levels <1 g/day and <0.5 g/day was significantly lower in the C4d-positive group compared to the C4d-negative group. The two groups did not differ regarding glomerular filtration rate and the need for renal replacement therapy. Older age and C4d positivity were independently associated with a lower likelihood of achieving proteinuria <0.5 g/day at 6 months. **Conclusions**: Glomerular capillary C4d positivity was associated with a more pronounced proteinuric phenotype and a reduced likelihood of early proteinuria remission. These findings support the clinical relevance of glomerular capillary C4d staining in relation to early proteinuria remission; however, larger prospective studies incorporating standardized semiquantitative C4d assessment and long-term follow-up are required before C4d can be considered a validated prognostic biomarker.

## 1. Introduction

Glomerular diseases are characterized by heterogeneous pathophysiological mechanisms and variable clinical courses and represent a major cause of chronic kidney disease. Even among patients with similar histopathological diagnoses, significant variability exists in terms of proteinuria, decline in renal function, and progression to renal replacement therapy (RRT) [1]. This heterogeneity suggests that underlying immunological and inflammatory mechanisms may influence clinical outcomes beyond conventional histopathological classifications [2]. Native glomerular diseases comprise a heterogeneous group of disorders with distinct pathogenic mechanisms, including immune complex-mediated diseases such as IgA nephropathy, lupus nephritis, membranous nephropathy, and membranoproliferative glomerulonephritis, as well as podocytopathies such as focal segmental glomerulosclerosis and minimal change disease [3,4]. Although these diseases differ substantially in their underlying pathogenic mechanisms, increasing evidence indicates that complement activation contributes to disease pathogenesis across several native glomerular diseases. Complement activation has also been implicated in diabetic kidney disease, while C4d deposition has been described in renal amyloidosis, although the mechanisms and pathological significance of complement involvement may differ among disease entities [5,6,7].

The complement system can be activated through the classical, lectin, or alternative pathways, ultimately leading to amplification of inflammation and tissue injury within the glomerulus [8]. In immune complex-mediated glomerular diseases, activation of the classical and lectin pathways by deposited immune complexes promotes glomerular inflammation and structural damage through complement-mediated effector mechanisms [9,10].

C4d, a degradation product generated during activation of the classical and lectin complement pathways, has long been recognized as a robust marker of antibody-mediated injury in kidney transplantation [11]. In renal allografts, C4d deposition within peritubular capillaries is widely used as a diagnostic hallmark of antibody-mediated rejection and reflects ongoing complement activation driven by donor-specific antibodies [12].

More recently, glomerular C4d deposition has also been investigated as a potential biomarker of complement-mediated injury in native kidney diseases. Several studies have reported associations between glomerular C4d positivity and disease severity or adverse renal outcomes in specific disease entities, particularly IgA nephropathy and lupus nephritis [13,14]. However, the available literature has largely focused on individual glomerular diseases and has used heterogeneous approaches to assess C4d deposition, including mesangial, segmental, or glomerular capillary wall staining patterns. Consequently, data specifically evaluating glomerular capillary wall C4d staining across a heterogeneous spectrum of native glomerular diseases remain limited. Therefore, the present study aimed to investigate the prevalence and clinical significance of glomerular capillary C4d staining in native kidney biopsies and to determine its association with short- and intermediate-term renal outcomes in patients with biopsy-proven glomerular diseases.

## 2. Materials and Methods

### 2.1. Patients

**Figure 1:**
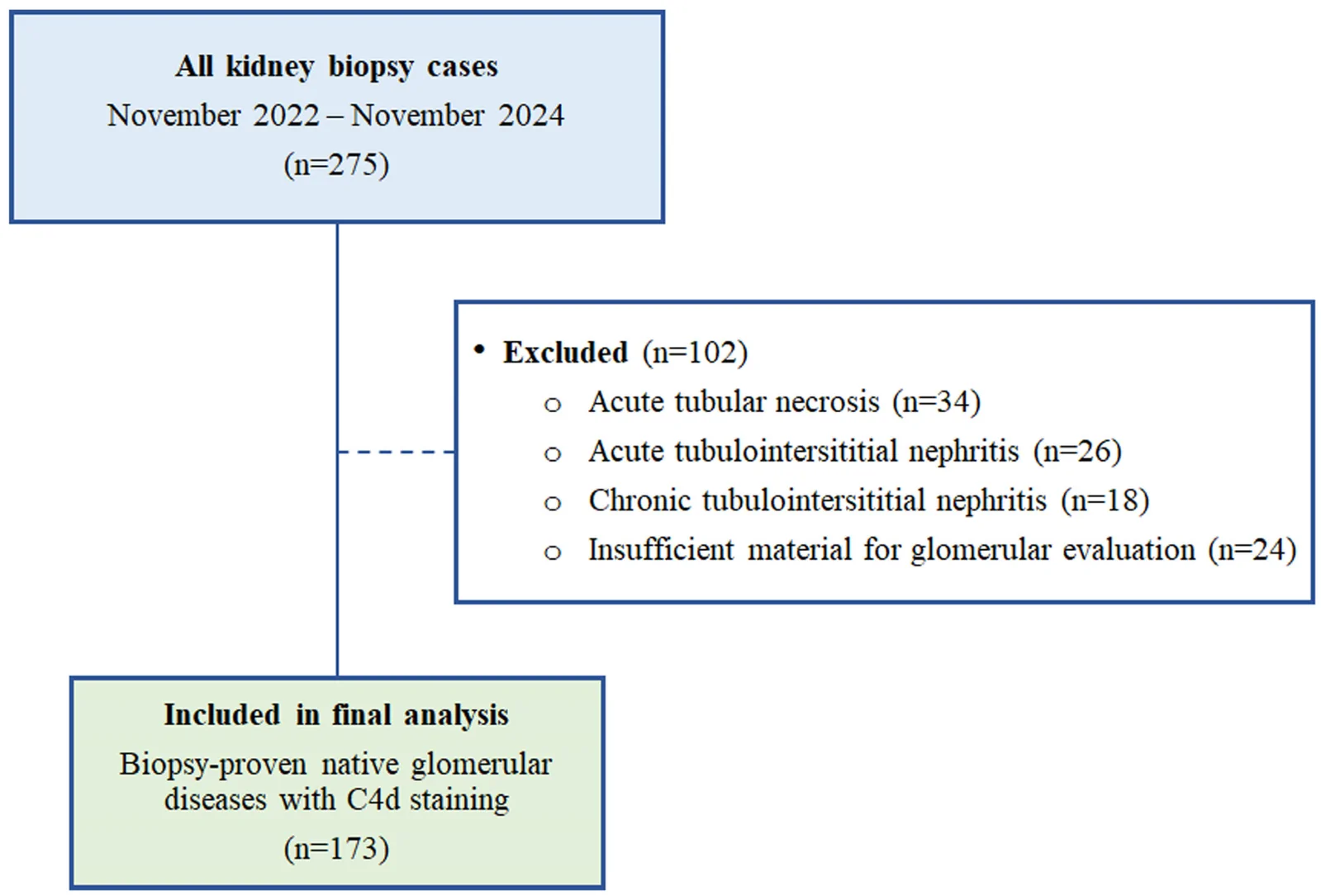
Study flow chart.

This single-center, retrospective study reviewed all native kidney biopsy cases performed on adult patients between November 2022 and November 2024. A total of 275 kidney biopsy reports were examined. Patients (*n* = 102) diagnosed with acute tubular necrosis (ATN), acute tubulointerstitial nephritis, or chronic tubulointerstitial nephritis, whose biopsy samples contained medulla or muscle tissue and were deemed insufficient for glomerular evaluation, were excluded from this study. Patients (*n* = 173) diagnosed with biopsy-proven glomerular disease and who underwent immunohistochemical C4d staining were included in this study (Figure 1).

Following the biopsy-based diagnosis of glomerular disease, patients were routinely followed in the nephrology outpatient clinic. Proteinuria was monitored using 24 h urinary protein measurements. Complete blood count, serum creatinine, serum albumin, and lipid profile were routinely assessed during outpatient follow-up as part of standard clinical care. Renal function was evaluated using serum creatinine and estimated glomerular filtration rate (eGFR), calculated using the 2021 CKD-EPI creatinine equation. These assessments reflected routine outpatient clinical practice at our institution and were not performed according to a study-specific monitoring protocol.

Follow-up assessments were performed at 6 months and 12–18 months after kidney biopsy. Of the 173 patients included in this study, 146 had documented clinical follow-up within the first 6 months, whereas follow-up data were unavailable for 27 patients during this period. Among the patients with documented follow-up, 20 initiated renal replacement therapy within the first 6 months and were therefore not considered evaluable for the proteinuria remission outcome. Accordingly, 126 patients were included in the 6-month proteinuria remission analysis. At the 12–18-month assessment, proteinuria follow-up data were available for 74 patients, whereas such data were unavailable for the remaining 99 patients. Owing to the retrospective design, the specific reasons for the absence of follow-up data could not be reliably determined from the available records.

Approval for this study was obtained from the Bursa City Hospital Scientific Research Ethics Committee (Date: 27 November 2024, No: 2024-20/8).

### 2.2. Pathological Evaluation and C4d Staining Protocol

All kidney biopsy specimens were independently evaluated by three experienced nephropathologists, and any discrepancies in interpretation were resolved by consensus review. The total number of glomeruli in each biopsy specimen was recorded as part of the histopathological evaluation.

During routine diagnostic evaluation, glomerular capillary C4d immunohistochemical staining was assessed qualitatively rather than using a semiquantitative scoring system. C4d positivity was defined as the presence of clear glomerular capillary wall staining in at least one evaluable glomerulus. In routine diagnostic practice, C4d-positive cases exhibited diffuse glomerular capillary wall staining. Accordingly, biopsy specimens were classified as C4d-positive or C4d-negative. Semiquantitative assessment of staining intensity, the proportion of positive glomeruli, and compartment-specific localization (glomerular versus tubular) was not routinely performed or systematically documented during routine diagnostic evaluation; therefore, these variables were unavailable for retrospective analysis. In a small number of biopsies from patients with anti-neutrophil cytoplasmic antibody (ANCA)-associated vasculitis, mesangial C4d positivity was also observed. However, the predefined study classification was based exclusively on glomerular capillary wall staining.

C4d immunohistochemical staining was performed using an anti-C4d antibody (Clone C4d204; ScyTek Laboratories, Logan, UT, USA; Catalog No. RA0025-C.1) at a dilution of 1:200 on a Ventana BenchMark Ultra automated staining platform (Ventana Medical Systems, Tucson, AZ, USA). Heat-induced antigen retrieval was performed using Cell Conditioning 1 (CC1) for 52 min, and immunoreactivity was visualized using the ultraView Universal DAB Detection Kit (Ventana Medical Systems, Tucson, AZ, USA). Each immunohistochemical staining run included a C4d-positive renal tissue as an external positive control in accordance with the routine quality assurance procedures of our pathology laboratory.

### 2.3. Statistical Analysis

Statistical analyses were performed using IBM SPSS Statistics for Windows, version 25.0 (IBM Corp., Armonk, NY, USA). The normality of continuous variables was examined using the Shapiro–Wilk test. Descriptive statistics were presented as numbers and percentages for categorical variables, and as mean, standard deviation, median, and interquartile range (IQR) for numerical variables. For numerical variables, the Mann-Whitney U test was used when the normality condition was not met for intergroup comparisons. For categorical variables, the chi-square test and Fisher’s Exact test were used for intergroup comparisons. The statistical significance level was accepted as *p* < 0.05.

For multivariable analyses, diagnostic categories were grouped into clinically and pathophysiologically relevant clusters to avoid overfitting and to preserve statistical power given the sample size. To identify risk factors associated with achieving proteinuria levels below 1 g/day or below 0.5 g/day at 6 months, variables were initially examined using univariate logistic regression analysis. Subsequently, those variables meeting the criterion of *p* < 0.25 were included in the multivariate logistic regression model.

## 3. Results

Kidney biopsy samples from 173 patients (60.7% male) aged 18–86 years were analyzed. General characteristics of the patients are shown in Table 1. Of the patients, 40.7% had hypertension, and 25.1% had no chronic disease. C4d positivity was detected in 66 (38.2%) of the biopsy samples. No statistically significant difference was found in general characteristics between C4d-positive and C4d-negative patients.

Viral serology (hepatitis B surface antigen [HBsAg], antibodies against hepatitis C virus [anti-HCV], and antibodies against human immunodeficiency virus [anti-HIV]) was assessed to exclude secondary causes of glomerulonephritis. Of the 165 patients tested, 5 were found to be HBsAg positive. None of the patients tested positive for anti-HCV or anti-HIV.

Baseline routine laboratory test results are summarized in Table 2. Low-density lipoprotein (LDL) levels were significantly higher, and albumin levels were lower in the C4d-positive group compared to the C4d-negative group. Proteinuria levels were also higher in the C4d-positive group.

The most frequent histopathological diagnosis was FSGS (26%), followed by membranous nephropathy (MN) (17.9%) and amyloid A (AA) amyloidosis (12.7%). When the diagnostic distribution of C4d-positive patients was evaluated, MN patients constituted about one-third (34.8%). This was followed by AA amyloidosis (21.2%) and lupus nephritis (LN) (10.6%). Of the C4d-positive patients, 72.7% were in the non-proliferative disease group (Table 3).

**Figure 2:**
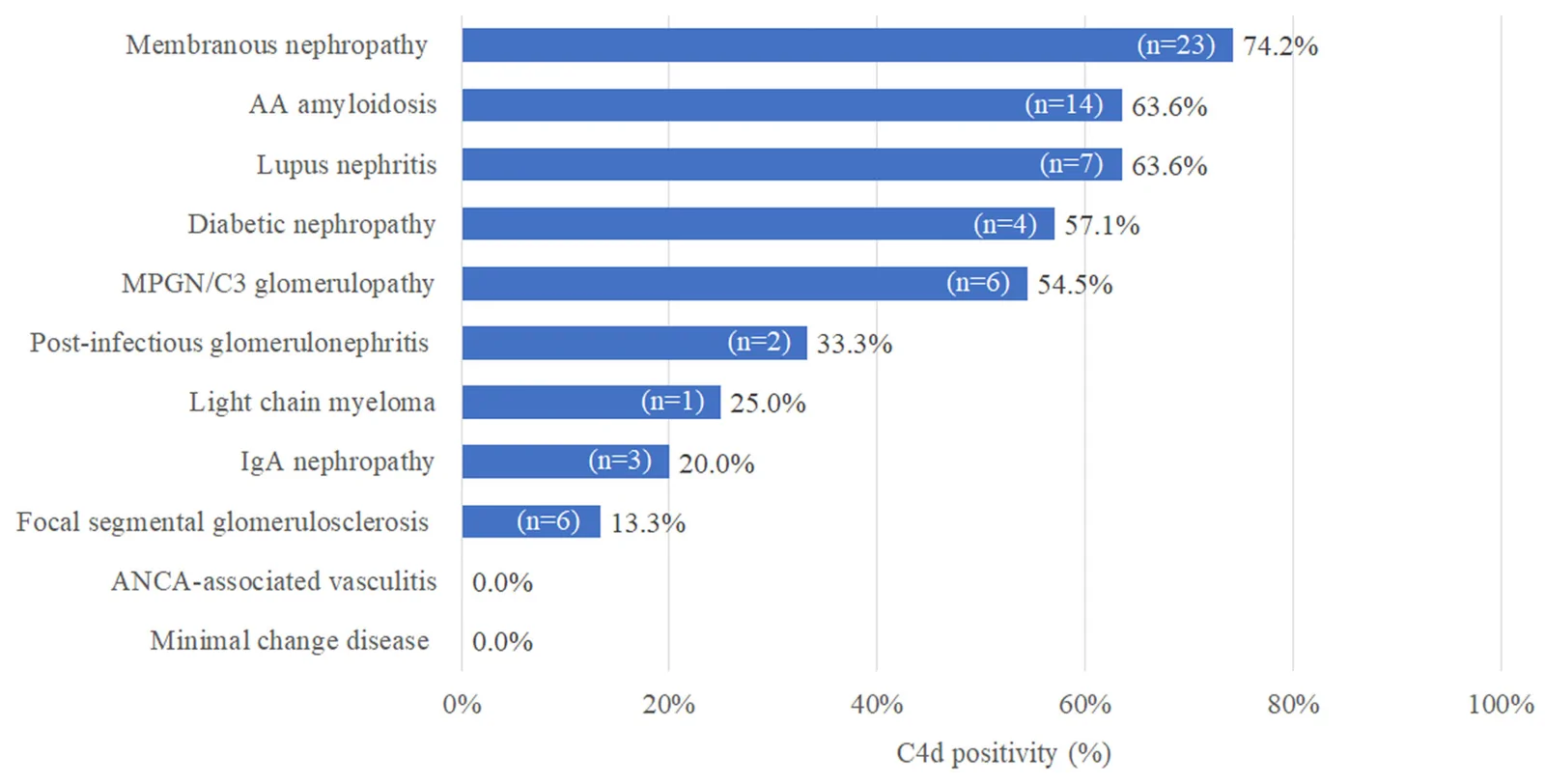
C4d positivity rates based on diagnosis. ANCA, Anti-neutrophil cytoplasmic antibodies; MPGN, membranoproliferative glomerulonephritis.

**Figure 3:**
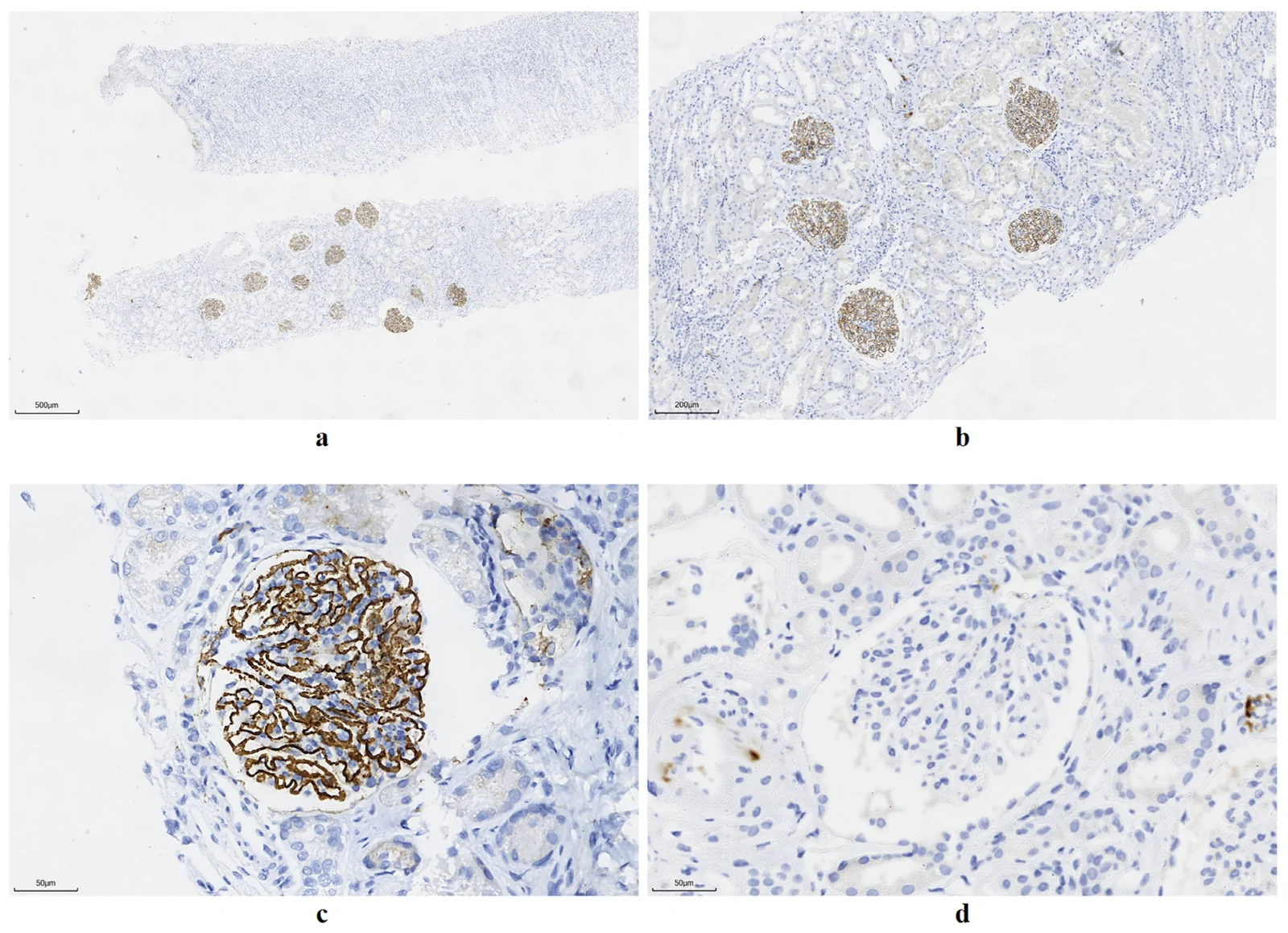
Representative immunohistochemical staining patterns of glomerular capillary C4d in native kidney biopsies. (**a**) Low-power view (original magnification ×40) demonstrating the overall distribution of glomerular C4d-positive staining. (**b**) Intermediate-power view (original magnification ×100) showing diffuse glomerular capillary C4d staining in multiple glomeruli. (**c**) High-power view (original magnification ×400) demonstrating diffuse linear C4d deposition along the glomerular capillary walls. (**d**) High-power view (original magnification ×400) showing the absence of glomerular capillary C4d staining. Scale bars: 500 μm (**a**), 200 μm (**b**), and 50 μm (**c**,**d**).

Based on histopathological diagnosis, the highest C4d positivity was observed in MN (74.2%), followed by AA amyloidosis (63.6%) and LN (63.6%). C4d positivity was detected in 13.3% of those diagnosed with FSGS. C4d positivity was not detected in ANCA-associated vasculitis and minimal change disease (MCD) (Figure 2). The C4d positivity rate was found to be 41.7% in the non-proliferative diagnosis group and 31.0% in the proliferative diagnosis group. Examples of C4d glomerular capillary staining are shown in Figure 3.

At 6 months, proteinuria values were available for 126 patients, estimated glomerular filtration rate (eGFR) values for 129 patients, and information regarding RRT for 146 patients. Proteinuria levels were higher in the C4d-positive group. At 6 months, the proportion of patients who achieved proteinuria levels < 1 g/day and <0.5 g/day was significantly lower in the C4d-positive group compared to the C4d-negative group. There was no difference between the two groups in terms of eGFR levels and the need for RRT (Table 4).

Proteinuria levels were available for 74 patients and eGFR levels for 81 patients at 12–18 months. Proteinuria levels were higher in the C4d-positive group. At 12–18 months, the proportion of patients who achieved proteinuria levels < 1 g/day was significantly lower in the C4d-positive group compared to the C4d-negative group. There was no difference between the two groups in terms of eGFR levels. (Table 5).

Two separate logistic regression models were constructed for proteinuria thresholds of <1 g/day and <0.5 g/day at 6 months. When factors associated with achieving proteinuria < 1 g/day at 6 months were examined using the univariate logistic regression model, the variables meeting the *p* < 0.25 criterion (age, C4d, 24-h urinary protein level, and diagnosis) were included in the multivariate logistic regression model. As a result, factors associated with achieving a proteinuria level of <1 g/day were identified as age, C4d positivity, and AA amyloidosis (Table 6).

When factors associated with achieving proteinuria < 0.5 g/day at 6 months were examined using the univariate logistic regression model, the variables meeting the *p* < 0.25 criterion (age and C4d) were included in the multivariate logistic regression model. As a result, factors associated with achieving proteinuria < 0.5 g/day were identified as age and C4d positivity (Table 7).

## 4. Discussion

C4d has long been considered a reliable marker of antibody-mediated rejection in the context of transplantation [15,16]. In recent years, the role of C4d in native kidney diseases has also been increasingly investigated. In many glomerular diseases, complement activation is one of the fundamental pathogenetic mechanisms, and this process can be reflected by C4d accumulation at the glomerular level [17]. Studies on C4d staining in native kidney diseases have largely focused on its diagnostic contribution, but its association with clinical course and prognosis has been evaluated in a limited number of studies. Therefore, it is not yet clear whether C4d accumulation is merely a diagnostic finding or whether it can be used as a biomarker reflecting disease activity and clinical outcomes. In this study, we aimed to evaluate the association of C4d positivity at the glomerular capillary level with clinical phenotype and renal outcomes, and to determine whether its association with proteinuria remission is independent of other clinical factors.

C4d glomerular staining has been considered a diagnostic marker, particularly in immune complex-mediated glomerular diseases [18]. Previous studies have demonstrated that C4d accumulation is observed at varying frequencies across different glomerular disease groups. It has been reported that C4d staining is particularly prominent in LN and membranoproliferative glomerulonephritis (MPGN) [6]. In addition, high levels of glomerular C4d accumulation have consistently been reported in MN [17,19]. The consistently high C4d positivity observed in LN, MN, MPGN, and post-infectious glomerulonephritis (PIGN) is thought to reflect abundant immune complex deposition together with activation of the classical and/or lectin complement pathways, which generate C4d as a stable complement split product [6,20]. This is consistent with previous studies suggesting that complement activation, particularly through the lectin pathway, plays an important role in the pathogenesis of MN [18]. In contrast, C4d reactivity appears to be more heterogeneous in diseases such as IgA nephropathy and MCD [21]. The relatively low frequency of C4d positivity observed in MCD may, at least in part, be explained by the absence or only minimal immune complex deposition and limited activation of the classical and lectin complement pathways, resulting in reduced C4d generation within the glomeruli [21,22], although this hypothesis requires further validation. Recent immunohistochemical studies have likewise demonstrated lower and more heterogeneous C4d expression in MCD compared with focal segmental glomerulosclerosis (FSGS), suggesting that C4d staining may have potential diagnostic utility in differentiating these two podocytopathies [23]. Nevertheless, the mechanisms underlying disease-specific differences in C4d deposition remain incompletely understood and warrant further investigation.

In a large series of 519 native kidney biopsies, Drachenberg et al. reported strong glomerular C4d staining in all MN cases, with a diffuse-global pattern in 100%, whereas 62% of MCD cases were negative and the remaining 38% showed only low-intensity mesangial staining [6]. Although C4d staining was detected in 97.4% of FSGS cases, it was predominantly segmental and localized to sclerotic lesions (83.2%), with diffuse-global staining in only 14.2%.

Similarly, Pradeep and Srinivas reported consistent glomerular basement membrane C4d deposition in MN, including class V LN, and immune complex-mediated MPGN, whereas mesangial and capillary wall staining was variable in FSGS, MCD, and other glomerular diseases [21]. Taken together, these studies provide a useful context for the disease-specific pattern observed in our cohort, characterized by high glomerular capillary C4d positivity in MN and lower positivity in FSGS and MCD. The apparent discrepancy in FSGS, particularly the high overall staining rate reported by Drachenberg et al. compared with the lower capillary positivity in our series, may reflect differences in staining localization, pathological positivity criteria, patient populations, disease subtype distributions, staining methodologies, and underlying complement activation mechanisms.

In FSGS, varying rates of C4d positivity have been reported, with some studies documenting relatively high frequencies [24]. However, in our series, C4d positivity was significantly lower in FSGS. This difference may be related to the heterogeneous nature of FSGS. Specifically, pathophysiological differences exist between primary and secondary FSGS subtypes, with hemodynamic and adaptive mechanisms being more dominant in secondary FSGS, leading to more limited immune complex and complement activation. Consequently, the relatively low C4d positivity observed in our cohort may reflect the higher proportion of secondary FSGS cases. However, because primary and secondary FSGS were not analyzed separately, this hypothesis could not be evaluated further and warrants future investigation.

At this point, it is important not only to consider the distribution of C4d accumulation among diseases, but also its relationship with clinical course and prognosis. The relationship between C4d positivity and prognosis has been evaluated in a limited number of studies, and a significant portion of the available data is based on specific disease subgroups, particularly IgA nephropathy. In IgA nephropathy, C4d accumulation has been reported to be associated with a more severe clinical and histopathological phenotype, and renal survival is worse in C4d-positive patients [17,25,26]. Zagorec et al. [27] reported that, among patients with primary IgA nephropathy, C4d-positive patients had higher blood pressure values, higher 24 h proteinuria, higher serum uric acid values, and lower eGFR values at the time of diagnosis, and had worse long-term renal outcomes. More recently, a contemporary observational cohort study by Viswanathan et al. further confirmed the prognostic significance of mesangial C4d deposition in IgA nephropathy, demonstrating that C4d positivity was independently associated with progression to kidney failure and provided incremental prognostic value beyond conventional clinicopathological parameters [28]. These findings collectively support the concept that C4d deposition reflects more aggressive disease biology and may serve as a useful prognostic biomarker in IgA nephropathy.

Outside IgA nephropathy, however, contemporary prognostic evidence remains limited. In glomerular diseases other than IgA nephropathy, the prognostic role of C4d has been defined to a more limited extent. Martin et al. [29] demonstrated that plasma C4d levels correlate with glomerular C4d accumulation in SLE patients. Furthermore, it has been reported that this marker may be a potential biomarker for distinguishing the presence of lupus nephritis and monitoring disease activity and clinical outcomes. In the present study, glomerular capillary C4d staining was evaluated qualitatively according to the routine diagnostic practice at our institution. Specifically, C4d positivity was defined as the presence of clear glomerular capillary wall staining in at least one evaluable glomerulus, and biopsy specimens were classified as C4d-positive or C4d-negative. Although semiquantitative assessment may provide additional pathological information, published studies have adopted heterogeneous approaches for evaluating glomerular C4d deposition. For example, Şahin et al. [30] evaluated staining intensity using a semiquantitative scoring system, Espinosa et al. [13] defined C4d positivity according to the proportion of positive glomeruli, Romero et al. [31] combined staining intensity with the percentage of positive glomeruli, whereas Satyam et al. [32] assessed C4d deposition semiquantitatively across multiple renal compartments. More recently, Zagorec et al. [27] also adopted a qualitative classification, defining cases as C4d-positive or C4d-negative based on the presence of glomerular capillary wall staining in at least one evaluable glomerulus, without applying a semiquantitative scoring system. Collectively, these studies illustrate the methodological heterogeneity that currently exists in the assessment of glomerular C4d deposition. Because our study was retrospective and based on routine pathology reports, semiquantitative assessment of staining intensity, the proportion of positive glomeruli, and compartment-specific localization was not available for analysis. This methodological limitation should be considered when interpreting our findings. Future prospective studies using standardized semiquantitative assessment methods are warranted to better define the association between glomerular C4d deposition and clinical outcomes.

Although mesangial C4d staining was not included in the predefined positivity criteria and was not systematically analyzed in our study, it was observed as a noteworthy finding in some ANCA-associated glomerulonephritis cases. Consistent with this observation, previous studies investigating the role of complement activation in ANCA-associated glomerulonephritis have shown the presence of C4d accumulation, but the distribution of this accumulation according to renal compartments has not been detailed [33,34]. This staining observed at the mesangial level may reflect local complement activation that can develop independently of immune complex accumulation, possibly suggesting a contribution of alternative or lectin pathway activation.

In our study, when the clinical implications of these histopathologically identified differences were evaluated, it was shown that C4d-positive patients were characterized by higher proteinuria levels, lower serum albumin values, and higher LDL cholesterol levels, and these differences were statistically significant. These findings support the association of C4d accumulation with a more pronounced nephrotic phenotype. These phenotypic differences were not limited to baseline clinical characteristics but were also reflected in early proteinuria outcomes. Indeed, in our study, proteinuria levels remained higher during follow-up, and rates of proteinuria remission were lower in C4d-positive patients.

Using a semiquantitative approach in 51 native kidney biopsies, Satyam et al. demonstrated a positive correlation between glomerular capillary wall C4d staining intensity and concurrent 24-h urinary protein excretion (*p* = 0.02) [32]. This finding supports the association observed in our cohort between glomerular capillary C4d positivity and a more pronounced proteinuric phenotype. However, whereas their cross-sectional study focused on concurrent proteinuria, our study extends these observations by evaluating the association between glomerular capillary C4d positivity and subsequent proteinuria remission at 6 months.

Multivariable analyses showed that glomerular capillary C4d positivity was independently associated with a lower likelihood of achieving early proteinuria remission at the 6-month follow-up. This finding suggests that C4d accumulation may not only reflect disease severity but may also be associated with a reduced likelihood of achieving early proteinuria remission. Since the primary objective of the present study was to evaluate early proteinuria remission, multivariable analyses were intentionally restricted to the 6-month follow-up. At the 12–18-month follow-up, the difference in achieving proteinuria <1.0 g/day remained statistically significant, whereas the difference for the more stringent remission threshold of <0.5 g/day was no longer significant. Although these findings suggest that the association between glomerular capillary C4d positivity and proteinuria remission persisted over time, they should be interpreted cautiously because these analyses were exploratory, included a substantially smaller number of patients with available follow-up, and were not supported by multivariable analyses. Although an eGFR reduction of ≥30% and the need for RRT were observed more frequently in C4d-positive patients, these differences did not reach statistical significance. Nevertheless, these findings may still be clinically relevant and should be further evaluated in larger prospective studies with standardized long-term follow-up. In our study, proteinuria remission was evaluated according to widely accepted thresholds in the literature, with ≤0.5 g/day representing complete remission and ≤1.0 g/day representing partial remission. Although no universally accepted threshold exists, these definitions are commonly used in glomerular disease studies, and lower residual proteinuria has consistently been associated with more favorable renal outcomes [35,36,37].

In our study, treatment approaches were heterogeneous, and both immunosuppressive and conservative therapies were selected according to underlying glomerular diagnosis, disease severity, and individual clinical characteristics rather than C4d status (Supplementary Table S1). Therefore, treatment variables were not incorporated into the multivariable models because adjustment for treatment would remain highly susceptible to confounding by indication and could potentially introduce additional bias rather than adequately control for it. Accordingly, treatment data are presented descriptively in Supplementary Table S1 to improve transparency regarding treatment distribution across the study cohort. Nevertheless, residual confounding related to treatment heterogeneity cannot be completely excluded and should be considered when interpreting the observed associations.

The major strengths of this study include the evaluation of glomerular C4d deposition in a relatively large real-world cohort encompassing a broad spectrum of native glomerular diseases, allowing assessment across diverse pathological entities. In addition, C4d immunohistochemical staining was performed using a standardized protocol, and all biopsy specimens were independently evaluated by three experienced nephropathologists with consensus review, enhancing the consistency and reliability of the pathological assessment. Furthermore, the integration of pathological findings with detailed clinical characteristics and early renal outcomes provides clinically relevant information regarding the association between glomerular C4d deposition and subsequent proteinuria remission.

Our study also has several limitations. First, as a retrospective single-center study, the generalizability of our findings may be limited. Second, treatment approaches were heterogeneous, and no standardized treatment protocol was applied across the study cohort. Because treatment allocation was primarily determined by the underlying glomerular diagnosis, disease severity, and individual clinical characteristics, residual confounding related to treatment heterogeneity and confounding by indication cannot be completely excluded despite multivariable adjustment. Third, C4d staining was assessed qualitatively as a binary variable (positive/negative) during routine diagnostic evaluation, without standardized semiquantitative assessment of staining intensity or the proportion of positive glomeruli. Therefore, quantitative analyses evaluating the relationship between staining intensity and clinical outcomes could not be performed. Finally, since C4d staining was evaluated exclusively at the glomerular capillary level, the absence of systematic assessment of mesangial, tubular, and vascular C4d deposition may have limited a more comprehensive evaluation of the intrarenal distribution of C4d. Future prospective studies integrating standardized semiquantitative assessment of glomerular C4d deposition with transcriptomic and other molecular profiling approaches are warranted to further elucidate the mechanisms underlying disease-specific patterns of C4d deposition and local complement activation, and to better define the diagnostic and prognostic significance of glomerular C4d deposition across native glomerular diseases.

## 5. Conclusions

This study demonstrates that glomerular capillary C4d positivity is associated with a more pronounced proteinuric phenotype and a lower likelihood of achieving proteinuria remission at the 6-month follow-up. This association remained significant after multivariable adjustment; however, prospective validation is required before glomerular capillary C4d can be considered a validated prognostic biomarker. The findings from the 12–18-month follow-up should also be interpreted cautiously because they were based on exploratory analyses involving a smaller number of patients. Overall, our findings support the clinical relevance of glomerular capillary C4d staining in relation to early proteinuria remission and provide a rationale for larger prospective studies incorporating standardized semiquantitative C4d assessment and long-term follow-up.

## Figures and Tables

**Table 1:** General characteristics of the patients.

	Total (*N* = 173)	C4d		*p*
		Negative (*N* = 107)	Positive (*N* = 66)	
**Age, years,** mean ± SD, median (IQR)	47.62 ± 16.28	47.44 ± 16.45	47.91 ± 16.11	0.793 ^1^
	47 (27)	45 (26)	50 (28)	
**Sex**, *n* (%)				
Male	105 (60.7)	63 (58.9)	42 (63.6)	0.534 ^2^
Female	68 (39.3)	44 (41.1)	24 (36.4)	
**Comorbidity**, *n* (%)				
*Cardiometabolic*				
Hypertension	68 (40.7)	40 (38.8)	28 (43.8)	0.530 ^2^
Diabetes mellitus	26 (15.6)	16 (15.5)	10 (15.6)	0.987 ^2^
Coronary artery disease	19 (11.4)	9 (8.7)	10 (15.6)	0.173 ^2^
Hypothyroidism	13 (7.8)	8 (7.8)	5 (7.8)	>0.99 ^3^
*Autoimmune/Inflammatory*				
Familial Mediterranean fever	4 (2.4)	1 (1)	3 (4.7)	0.158 ^3^
Psoriasis	1 (0.6)	0	1 (1.6)	0.383 ^3^
Rheumatoid arthritis	8 (4.8)	5 (4.9)	3 (4.7)	>0.99 ^3^
Ankylosing spondylitis	1 (0.6)	1 (1)	0	>0.99 ^3^
Sjögren’s syndrome	2 (1.2)	1 (1)	1 (1.6)	>0.99 ^3^
Systemic lupus erythematosus	4 (2.4)	1 (1)	3 (4.7)	0.158 ^3^
Myasthenia gravis	1 (0.6)	1 (1)	0	>0.99 ^3^
*Others*				
Chronic obstructive pulmonary disease	4 (2.4)	2 (1.9)	2 (3.1)	0.638 ^3^
Asthma	7 (4.2)	5 (4.9)	2 (3.1)	0.709 ^3^
Tuberculosis	1 (0.6)	0	1 (1.6)	0.383 ^3^
Malignancy	3 (1.8)	3 (2.9)	0	0.287 ^3^
Parkinson disease	1 (0.6)	0	1 (1.6)	0.383 ^3^
Epilepsy	1 (0.6)	1 (1)	0	>0.99 ^3^
Thalassemia	1 (0.6)	0	1 (1.6)	0.383 ^3^
Benign prostatic hyperplasia	3 (1.8)	2 (1.9)	1 (1.6)	>0.99 ^3^
**No chronic disease**, *n* (%)	42 (25.1)	27 (26.2)	15 (23.4)	0.688 ^3^

^1^ Mann–Whitney U test, ^2^ Pearson Chi-Square test, ^3^ Fisher’s Exact test.

**Table 2:** Baseline laboratory test results.

	Total (*N* = 173)	C4d		*p*
		Negative (*N* = 107)	Positive (*N* = 66)	
Hemoglobin, g/dL	12.4 (4.4)	12.9 (4.5)	12.3 (3.83)	0.945 ^1^
ESR, mm/h	44 (50)	37 (46)	43.5 (43.5)	0.324 ^1^
Glucose, mg/dL	94 (21)	96 (20)	91 (17)	0.272 ^1^
Total cholesterol, mg/dL	223 (154)	208 (121)	267.5 (178)	0.052 ^1^
LDL, mg/dL	135 (117)	127.5 (94)	172.5 (142)	**0.024 ^1^**
HDL, mg/dL	42 (19)	42 (20)	41.5 (17)	0.843 ^1^
Triglycerides, mg/dL	178 (150)	168 (153)	189.5 (154)	0.456 ^1^
Albumin, g/L	31.3 (14.5)	34 (14.9)	28.05 (11.43)	**0.008 ^1^**
AST, IU/L	17 (8)	17 (9)	17 (7)	0.612 ^1^
ALT, IU/L	15 (11)	16 (12)	14 (9)	0.361 ^1^
TSH, mIU/L	2.29 (2.48)	2.12 (2.22)	2.35 (2.37)	0.151 ^1^
CRP, mg/L	5.3 (17.2)	4.9 (20.9)	5.5 (10.9)	0.852 ^1^
Creatinine, mg/dL	1.3 (1.65)	1.3 (1.67)	1.11 (1.07)	0.681 ^1^
Uric acid, mg/dL	6.1 (1.9)	6.1 (1.92)	5.9 (2.02)	0.568 ^1^
eGFR, mL/min/1.73 m^2^	62.5 (80.4)	63.4 (83)	73.7 (64.1)	0.740 ^1^
Proteinuria, mg/day	4000 (4918.5)	3460 (3941)	5729 (5474.8)	**<0.001 ^1^**
Hematuria, *n* (%)	86 (49.7)	58 (54.2)	28 (42.4)	0.132 ^2^
C3, g/L	1.31 ± 0.42	1.35 ± 0.39	1.25 ± 0.46	0.168 ^4^
C4, g/L	0.3 (0.18)	0.3 (0.18)	0.29 (0.18)	0.659 ^1^

Data are expressed as mean ± standard deviation, median (IQR), and *n* (%). ^1^ Mann–Whitney U test, ^2^ Pearson Chi-Square test, ^4^ Independent Samples test. ALT, alanine transaminase; AST, aspartate transaminase; CRP, C-reactive protein; ESR, erythrocyte sedimentation rate; eGFR, estimated glomerular filtration rate; HDL, high-density lipoprotein; LDL, low-density lipoprotein; TSH, thyroid-stimulating hormone. Bold values indicate statistical significance (*p* < 0.05).

**Table 3:** Histopathological diagnoses and C4d staining status.

Diagnosis	Total (*N* = 173)	C4d	
		Negative (*N* = 107)	Positive (*N* = 66)
	*n* (%)	*n* (%)	*n* (%)
Focal segmental glomerulosclerosis (FSGS)	45 (26.0)	39 (36.4)	6 (9.1)
Membranous nephropathy (MN)	31 (17.9)	8 (7.5)	23 (34.8)
AA amyloidosis	22 (12.7)	8 (7.5)	14 (21.2)
ANCA-associated vasculitis ^1^	15 (8.7)	15 (14)	0 (0.0)
IgA nephropathy	15 (8.7)	12 (11.2)	3 (4.5)
Membranoproliferative glomerulonephritis (MPGN)/C3 glomerulopathy ^2^	11 (6.4)	5 (4.7)	6 (9.1)
Lupus nephritis	11 (6.4)	4 (3.7)	7 (10.6)
Diabetic nephropathy	7 (4.0)	3 (2.8)	4 (6.1)
Minimal change disease (MCD)	6 (3.5)	6 (5.6)	0 (0.0)
Post-infectious glomerulonephritis (PIGN)	6 (3.5)	4 (3.7)	2 (3.0)
Light chain myeloma	4 (2.3)	3 (2.8)	1 (1.5)

^1^ C4d positivity was primarily defined by glomerular capillary staining; mesangial C4d deposition observed in a limited number of ANCA-associated vasculitis cases was reported descriptively as a disease-specific pattern. ^2^ MPGN and C3 glomerulopathy were analyzed together due to the limited number of cases in each subgroup. ANCA, anti-neutrophil cytoplasmic antibody; MPGN, membranoproliferative glomerulonephritis. Percentages may not total 100% due to rounding.

**Table 4:** Renal outcomes of patients at 6 months according to C4d status.

	C4d		*p*
**Proteinuria Outcomes**	**Negative** **(*N* = 81)**	**Positive** **(*N* = 45)**	
Proteinuria at baseline, mg/day	3460 (3862.5)	6055 (6198)	**<0.001 ^a^**
Proteinuria at 6 months, mg/day	832 (1693)	2700 (5129)	**<0.001 ^a^**
Δ Proteinuria, mg/day	↓ 1496 (3855)	↓ 2454 (3582)	0.288 ^a^
≥50% reduction in proteinuria, *n* (%)	47 (58.0)	22 (52.4)	0.550 ^b^
Proteinuria < 1.0 g/day at 6 months, *n* (%)	50 (61.7)	14 (33.3)	**0.003 ^b^**
Proteinuria < 0.5 g/day at 6 months, *n* (%)	30 (37.0)	8 (18.6)	**0.034 ^b^**
**Kidney Function Outcomes**	**Negative** **(*N* = 83)**	**Positive** **(*N* = 46)**	
eGFR at baseline, mL/min/1.73 m^2^	70 (69.5)	86.35 (57.5)	0.637 ^a^
eGFR at 6 months, mL/min/1.73 m^2^	73 (66)	76.5 (63)	0.696 ^a^
Δ eGFR, mL/min/1.73 m^2^	↓ 1.5 (18)	0.65 (15)	0.916 ^a^
≥30% decline in eGFR, *n* (%)	7 (8.4)	5 (11.6)	0.542 ^c^
**Hard Outcome**	**Negative** **(*N* = 93)**	**Positive** **(*N* = 53)**	
RRT, *n* (%)	10 (10.8)	10 (18.9)	0.170 ^b^

Data are expressed as median (IQR) or *n* (%). ^a^: Mann–Whitney U test, ^b^: Pearson Chi-Square test, ^c^: Fisher’s Exact test. Δ: The value obtained by subtracting the baseline value from the value of the 6th month. ↓: It indicates that the relevant value has decreased. eGFR, estimated glomerular filtration rate; RRT, renal replacement therapy. Bold *p*-values indicate statistical significance (*p* < 0.05).

**Table 5:** Long-term renal outcomes at 12–18 months according to C4d status.

	C4d		*p*
**Proteinuria Outcomes**	**Negative** **(*N* = 46)**	**Positive** **(*N* = 28)**	
Proteinuria at baseline, mg/day	3483.5 (3382.75)	6877 (5232.75)	**<0.001 ^a^**
Proteinuria at 12–18 months, mg/day	612 (1474)	1833 (5550)	**0.031 ^a^**
Δ Proteinuria, mg/day	↓ 1849.5 (3453.5)	↓ 3736.5 (6434.8)	**0.038 ^a^**
≥50% reduction in proteinuria, *n* (%)	29 (63.0)	17 (60.7)	0.841 ^b^
Proteinuria < 1.0 g/day at 12–18 months, *n* (%)	31 (67.4)	10 (35.7)	**0.008 ^b^**
Proteinuria < 0.5 g/day at 12–18 months, *n* (%)	19 (41.3)	8 (28.6)	0.270 ^b^
**Kidney Function Outcomes**	**Negative** **(*N* = 51)**	**Positive** **(*N* = 30)**	
eGFR at baseline, mL/min/1.73 m^2^	80 (63.9)	87.2 (59)	0.980 ^a^
eGFR at 12–18 months, mL/min/1.73 m^2^	65 (63)	72 (56)	0.891 ^a^
Δ eGFR, mL/min/1.73 m^2^	↓ 5 (14.4)	↓ 11.8 (28)	0.304 ^a^
≥30% decline in eGFR, *n* (%)	7 (14.0)	8 (26.7)	0.160 ^b^

Data are expressed as median (IQR) or *n* (%). ^a^: Mann–Whitney U test, ^b^: Pearson Chi-Square test. Δ: The value obtained by subtracting the baseline value from the value at 12–18 months. ↓: It indicates that the relevant value has decreased. eGFR, estimated glomerular filtration rate. Bold *p*-values indicate statistical significance (*p* < 0.05).

**Table 6:** Factors associated with achieving proteinuria < 1 g/day at 6 months.

	Univariate Logistic Regression Model		Multivariate Logistic Regression Model	
	OR (95% CI)	*p*	OR (95% CI)	*p*
Age	0.97 (0.94–0.99)	**0.007**	0.97 (0.94–0.99)	**0.014**
C4d (Positive)	0.31 (0.14–0.68)	**0.003**	0.34 (0.13–0.84)	**0.020**
24-h urine protein	1 (1–1)	**0.043**		
eGFR	1 (0.99–1.01)	0.377		
Diagnosis				
Membranous nephropathy	0.27 (0.11–0.69)	**0.007**	0.50 (0.17–1.445)	0.202
AA amyloidosis	0.10 (0.02–0.5)	**0.005**	0.13 (0.03–0.67)	**0.015**

OR, odds ratio; CI, confidence interval. The “negative” category was used as the reference category for the C4d variable, and the “other glomerular diseases” category was used as the reference category for the diagnosis variable. Bold *p*-values indicate statistical significance (*p* < 0.05).

**Table 7:** Factors associated with achieving proteinuria < 0.5 g/day at 6 months.

	Univariate Logistic Regression Model		Multivariate Logistic Regression Model	
	OR (95% CI)	*p*	OR (95% CI)	*p*
Age	0.96 (0.93–0.99)	**0.002**	0.96 (0.93–0.98)	**0.002**
C4d (Positive)	0.39 (0.16–0.95)	**0.038**	0.34 (0.13–0.86)	**0.023**
24-h urine protein	1 (1–1)	0.830		
eGFR	1.01 (0.99–1.02)	0.286		
Diagnosis				
Membranous nephropathy	0.39 (0.14–1.15)	0.089		
AA amyloidosis	0	0.998		

OR, odds ratio; CI, confidence interval. The “negative” category was used as the reference category for the C4d variable, and the “other glomerular diseases” category was used as the reference category for the Diagnosis variable. Bold *p*-values indicate statistical significance (*p* < 0.05).

## Data Availability

The raw data supporting the conclusions of this article will be made available by the authors on request.

## Supplementary Materials

The following supporting information can be downloaded at: https://www.mdpi.com/article/10.3390/diagnostics16172717/s1, Table S1: Treatments received during the first 6 months.

## Abbreviations

The following abbreviations are used in this manuscript:

AA	Amyloid A
ALT	Alanine transaminase
ANCA	Anti-neutrophil Cytoplasmic Antibody
anti-HIV	Antibodies against human immunodeficiency virus
AST	Aspartate transaminase
ATN	Acute tubular necrosis
CRP	C-reactive protein
eGFR	Estimated glomerular filtration rate
ESR	Erythrocyte sedimentation rate
FSGS	Focal segmental glomerulosclerosis
HBsAg	Hepatitis B surface antigen
HDL	High-density lipoprotein
IHC	Immunohistochemistry
IQR	Interquartile range
LDL	Low-density lipoprotein
LN	Lupus nephritis
MCD	Minimal change disease
MN	Membranous nephropathy
MPGN	Membranoproliferative glomerulonephritis
PIGN	Post-infectious glomerulonephritis
RRT	Renal replacement therapy
TSH	Thyroid stimulating hormone
