# Supplementary material for: Glomerular Capillary C4d Staining in Native Kidney Biopsies: Associations with Proteinuric Phenotype and Reduced Likelihood of Early Proteinuria Remission
Source: Diagnostics (Basel). 2026 Aug 26;16(17):2717. doi: 10.3390/diagnostics16172717 (PMC13565186; doi:10.3390/diagnostics16172717)
Supplement: Supplementary file 1 [file diagnostics-16-02717-s001.zip › diagnostics-4444169-supplementary.pdf]

Supplementary Table S1. Treatments received during the first 6 months

	Total	C4d	
		**Negative (n=98)**	**Positive (n=60)**
**Immunosuppressive therapy**	n (%)	n (%)	n (%)
Corticosteroids	82 (51.9)	53 (54.1)	29 (48.3)
Mycophenolate mofetil	19 (12)	7 (7.1)	12 (20.0)
Cyclophosphamide	15 (9.5)	11 (11.2)	4 (6.7)
Rituximab	5 (3.2)	2 (2.0)	3 (5.0)
Cyclosporine	23 (14.6)	15 (15.3)	8 (13.3)
Tacrolimus	3 (1.9)	0	3 (5.0)
Azathioprine	2 (1.3)	2 (2.0)	0 (0.0)
**Conservative therapy**			
RAS blockade (ACEi/ARB)	138 (87.3)	85 (86.7)	53 (88.3)
SGLT2 inhibitors	30 (19.0)	18 (18.4)	12 (20.0)
Finerenone	14 (8.9)	10 (10.2)	4 (6.7)

ACEi, angiotensin-converting enzyme inhibitor; ARB, angiotensin II receptor blocker; SGLT2, sodium-glucose cotransporter 2, RAS, renin–angiotensin system
